# Intranasal Xylitol for the Treatment of COVID-19 in the Outpatient Setting: A Pilot Study

**DOI:** 10.7759/cureus.27182

**Published:** 2022-07-23

**Authors:** Evangelina Soler, Amanda de Mendoza, Víctor I Cuello, Maria G Silva-Vetri, Zoilangel H Núñez, Ramsés G Ortega, Syed A Rizvi, Marcos Sanchez-Gonzalez, Gustavo Ferrer

**Affiliations:** 1 Department of Pulmonolgy, Clinica Corazones Unidos, Santo Domingo, DOM; 2 Department of Research, Universidad Nacional Pedro Henríquez Ureña School of Medicine, Santo Domingo, DOM; 3 Graduate Medical Education and Biomedical Sciences, Larkin Hospital and University, Miami, USA; 4 Graduate Medical Education, Larkin Community Hospital, South Miami, USA; 5 Pulmonary Critical Care, Aventura Hospital and Medical Center, Aventura, USA

**Keywords:** sars-cov-2, saline, anosmia, intranasal therapies, nasal hygiene, covid-19, xylitol

## Abstract

It is well known that acute COVID-19 infection can present with a variety of symptoms, including fever, cough, rhinitis, loss of taste, and the cardinal sign of loss of smell (anosmia). Recently, nasal irrigations with saline and other agents have shown promise for the treatment of COVID-19. Xylitol has been shown to display virucidal effects against SARS-CoV-2. This study aimed to examine the efficacy of xylitol as an adjunct treatment for COVID-19 in an outpatient setting. In a randomized controlled double-blinded fashion, a total of 50 participants (F=30) consented to participate in this study. It was a population of 18 to 65 years of age, with polymerase chain reaction confirmed for SARS-COV-2 by nasopharyngeal swab, less than three days from the start of symptoms. This study's primary endpoint was time to clinical recovery, defined as the change from baseline to end of treatment in COVID-19 symptoms. Outcome variables were the changes in visual analog scale (VAS) and daily symptoms score (DSS) on Days 1-7, 14, and 28 after the initiation of the 14-day treatment. There were no differences between the treatment groups in any demographic and subject characteristics-related variables, including vaccination status. None of the patients were hospitalized, or required emergency visits in addition to no adverse reactions were reported. There were no statistically significant interactions found for VAS (P=0.124), DSS (P=0.448), and sense of smell (P=0.667). The proportion of patients reporting nasal congestion was higher (X^2^=5.05; P=0.025) in the xylitol (XYL) group (73.1%) vs. the saline (SAL) group (41.7%) on Day 4, and on Day 7 (X^2^=5.72; P=0.017) XYL group (50.0%) vs. SAL group (17.4%). During Day 28 a total of two patients in the SAL group had anosmia vs. no patients with anosmia in the XYL group, although this difference did not reach statistical significance (X^2^=5.72; P=0.133). Results demonstrate that both xylitol and saline were equally effective in decreasing the time of symptom resolution and preventing hospitalizations, yet, persistent anosmia was only seen in the SAL group. Intranasal xylitol might play a pivotal role in preventing persistent olfactory abnormalities in post-COVID-19 patients.

## Introduction

The emergence of severe acute respiratory syndrome coronavirus 2 (SARS-CoV-2), the causative agent of the coronavirus disease 2019 (COVID-19), was declared a pandemic by the World Health Organization (WHO) on March 11, 2020. SARS-CoV-2 has infected more than 435 million people and caused nearly six million deaths in 188 countries [1]. COVID-19 is a biphasic clinical syndrome characterized by an initial viremic phase (one to seven days) followed by a hypersensitivity-like hyperinflammatory state (>8 days) mainly driven by mast cell histamine degranulation leading to a cytokine storm [2-5]. Intense efforts have been made worldwide to establish effective therapies and develop vaccines and other treatments for seriously ill patients. However, we are still investigating more alternatives to address this disease, particularly during the early stages of infection [6]. As a simple but potentially powerful tool in our medical arsenal, the addition of appropriate nasal hygiene practices with substances that can neutralize the virus could be another turning point in our fight against COVID-19 [7]. This is worth mentioning as the cells of the nasal epithelium have the highest percentage of angiotensin-converting enzyme 2 (ACE2) receptors, the gateway of (SARS-CoV-2) [8-10].

It is well known that acute COVID-19 infection can present with a variety of symptoms, among them, the most frequent are fever, cough, rhinitis, and loss of taste (ageusia) and smell (anosmia) [11-14]. Similarly, there are less frequent symptoms such as headache, abdominal pain, vomiting, diarrhea, sore throat, shortness of breath, conjunctivitis, swelling of lymph nodes, and drowsiness [14]. Considering that the nasal epithelium cells have the highest percentage of ACE2-expressing hair cells in the proximal airways, it is plausible to suggest that the addition of nasal disinfection practices with nasal sprays containing xylitol could be a candidate agent for providing effective preventive and therapeutic modalities against COVID-19. Recently, nasal sprays containing the xylitol (sugar alcohol and a sweetener) have been proposed to counteract the stage of greater virulence of the virus at the beginning of the disease (one to four days) to prevent the progression of the disease the infection and therefore the possible complications [15].

Xylitol has been shown to display virucidal effects against SARS-CoV-2 and with the addition of hypertonic saline and hyaluronate may shorten the viral shedding duration in asymptomatic COVID-19 positive subjects [16, 17]. Xylitol, with antimicrobial and anti-inflammatory properties, has been shown effective in improving chronic rhinitis as well as important microbiota and immunological modulatory effects [18-21]. Xylitol has been reported to have multiple health benefits as well as is generally safe and well-tolerated for most adults in doses up to 35 grams per day and up to 20 grams per day in children [22, 23]. Considering the pathophysiology of COVID-19, it is crucial to treat the initial symptoms as a therapeutic intervention for preventing the more severe complications. Therefore, this study aimed to examine the efficacy of xylitol as an adjunct treatment for COVID-19 in an outpatient setting. We hypothesized that patients treated with the xylitol nasal spray would have a faster clinical recovery as recorded by a reduction in symptoms from baseline to the end of treatment.

## Materials and methods

Patients

A total of 50 participants (F=30) consented to participate in this study. It was a population of 18 to 65 years of age, of both sexes, with polymerase chain reaction tests (PCR) confirming for SARS-COV-2 by nasopharyngeal swab, less than three days from the start of symptoms. Cases of severe symptomatology were excluded from the study as well as those with known hypersensitivity to one of the constituents, particularly xylitol, pregnant or breastfeeding women, and those with an impediment to using the spray correctly. The study proposal was reviewed and approved by the National Council of Bioethics in Health (CONABIOS) under code 036-2020 in Santo Domingo, Dominican Republic also registered as NCT04610801.

Study design

A randomized, double-blind controlled trial was developed as the choice of design. Randomization and matching were performed by someone not associated with the care or assessment of the patients by means of a computer-generated random number table (with a 20% random element) using an allocation ratio of 1:1 as previously described [24]. Patients were randomized to treatment with xylitol intranasal (XYL) and intranasal saline (NaCl 0.9%) as control (SAL). The xylitol spray consisted of purified water, 11% Pure Xylitol (Shandon Lujian, Shandong, China), 0.85% NaCL (Saline), and 0.20% grapefruit seed extract (GSE; Chemie Research & Manufacturing Co., Casselberry, FL, USA). Both treatments were administered through a nasal spray pump dispensing a volume of 140 µL. Two pumps of XYL or SAL were administered per nostril every three hours while awake during the first 72 hours. After that, two treatment applications were administered per nostril every six hours up to four times per day. Patients attended an outpatient pulmonology consultation and agreed to comply with the treatment for 14 continuous days. The daily symptomatology was monitored daily for the first seven days, then on Days 14 and 28. On the day of enrollment, patients were instructed on how to report the different scales, the importance of treatment adherence, and how to use the nasal spray correctly. All subjects were following the standard COVID-19 treatment protocol as established by the Government of the Dominican Republic [25]. The study flow chart is summarized in Table 1.

**Table 1:** Study flow chart PCR: polymerase chain reaction

Activity	Treatment												Follow-up	
Schedule (day)	1	2	3	4	5	6	7	8	9	10		14	28	
Study visits	1						2			3		4	5	
Contact study center	X													
Randomization	X													
Informed consent	X													
Inclusion and exclusion criteria	X													
Demographic data	X													
Temperature measurement	X						X			X		X		
Urine pregnancy test	X													
Pulse oximetry	X		X		X		X					X		
Sampling nasopharyngeal swabs	X						X					X		
PCR	X						X					X		
Assessment of subject status	X	X	X	X	X	X	X	X	X	X		X	X	
Safety assessment	X	X	X	X	X		X			X		X	X	
Tretament administration	X-------------------------------------------------------------------X													
Final assessment													X	

Outcomes and scales

In order to monitor symptoms related to the severity of the disease, various instruments were applied. This study's primary endpoint was time to clinical recovery, defined as the change from baseline to end of treatment in COVID-19 [11]. Briefly, the visual analog scale (VAS) was used as previously described on a scale of 1-10 was offered to represent "0 = no symptoms" and "10 = worst of symptoms" [26]. Clinical practice guidelines have suggested a classification in which "mild" AR = 0 to 3 cm, "moderate" AR = 3.1 to 7 cm, and "severe" AR = 7.1 to 10. The daily symptom assessment scale (DSS) on the scale (0-3) was used to confirm; 0 - none (No symptoms), 1 - light symptoms: minimum of respiratory symptoms or asymptomatic with positive PCR, 2 - light symptoms: respiratory symptoms such as cough, nasal obstruction, fever, malaise without desaturation, 3 - moderate to severe respiratory symptoms: hypoxia (SpO2 <88% is not corrected with oxygen at 2lts, in addition, severe tachypnea (if presented they should be excluded from the study). Analysis of olfactory function: (0 - normal; 1 - slightly damaged; 2 - moderately damaged; 3 - away). The presence of anosmia was also collected. The safety assessment was based on the patients' adverse effects and as evaluated by the physician. All serious adverse events and suspected drug-related hypersensitivity reactions were recorded.

Statistical analyses

Statistical analyses were performed using SPSS Version 26.0 (IBM Corp., Armonk, NY) to calculate descriptive and inferential statistics. Independent samples t-tests were used to compare the groups (XYL vs. SAL) in continuous variables, whereas chi-square analyses were used for categorical variables. The effects of XYL and SAL on COVID-19 symptoms were evaluated using a 9 X 2 repeated measures ANOVA (analysis of variance) with Bonferroni alfa adjustment for time effects from Day 1: time (Day 1 vs. Day 2 vs. Day 3 vs. Day 4 vs. Day 5 vs. Day 6 vs. Day 7 vs. Day 14 vs. Day 28) x treatment (XYL vs. SAL). 

## Results

**Figure 1:**
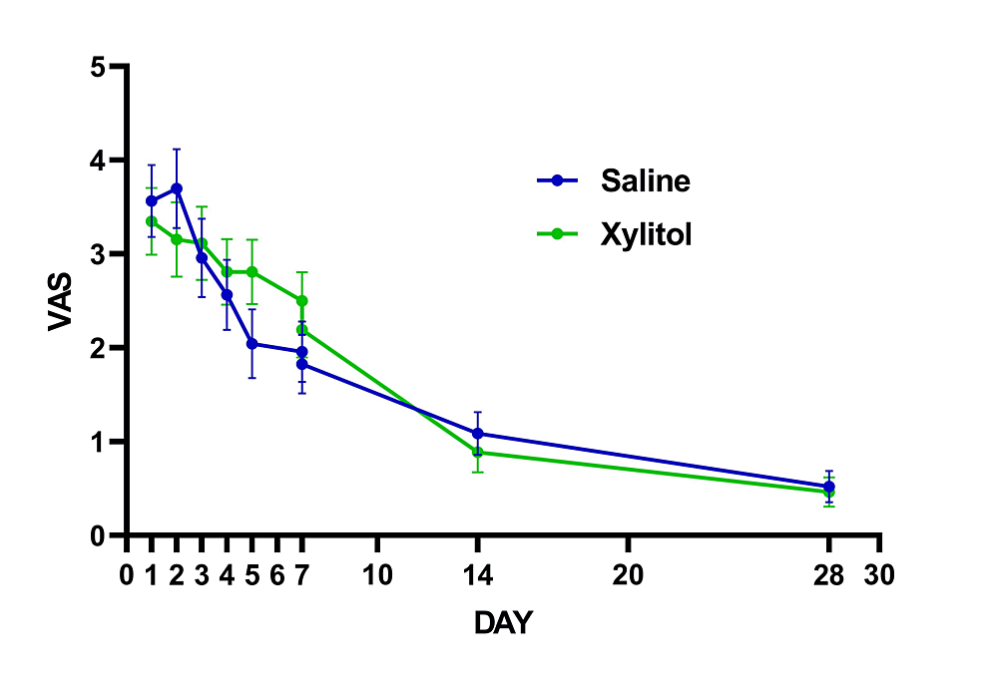
Responses in COVID-19 symptoms using the visual analog scale (VAS).

**Figure 2:**
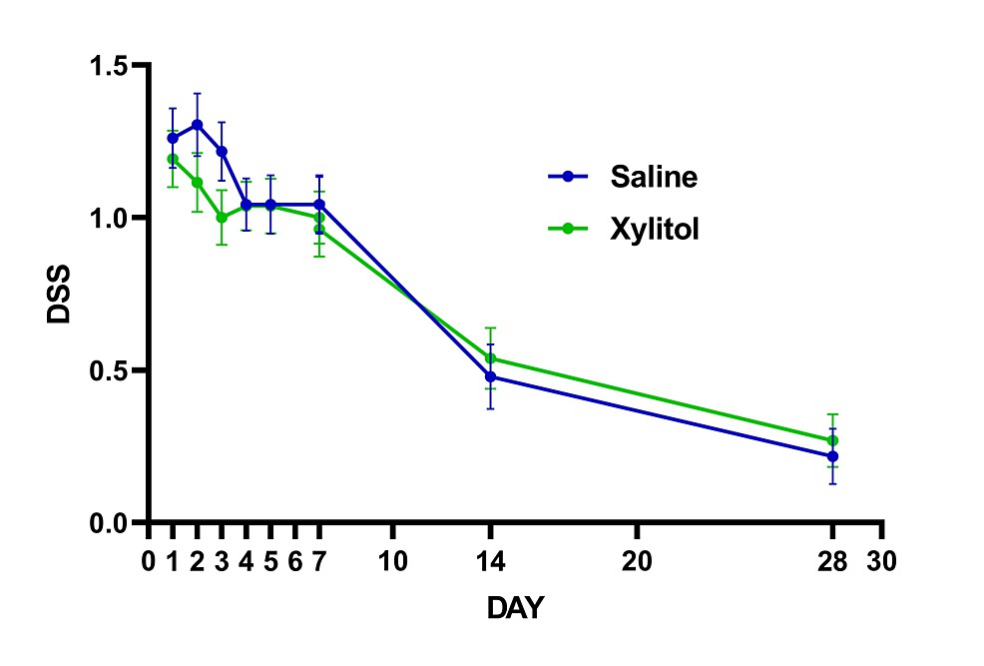
Responses in daily symptoms scores (DSS)

**Figure 3:**
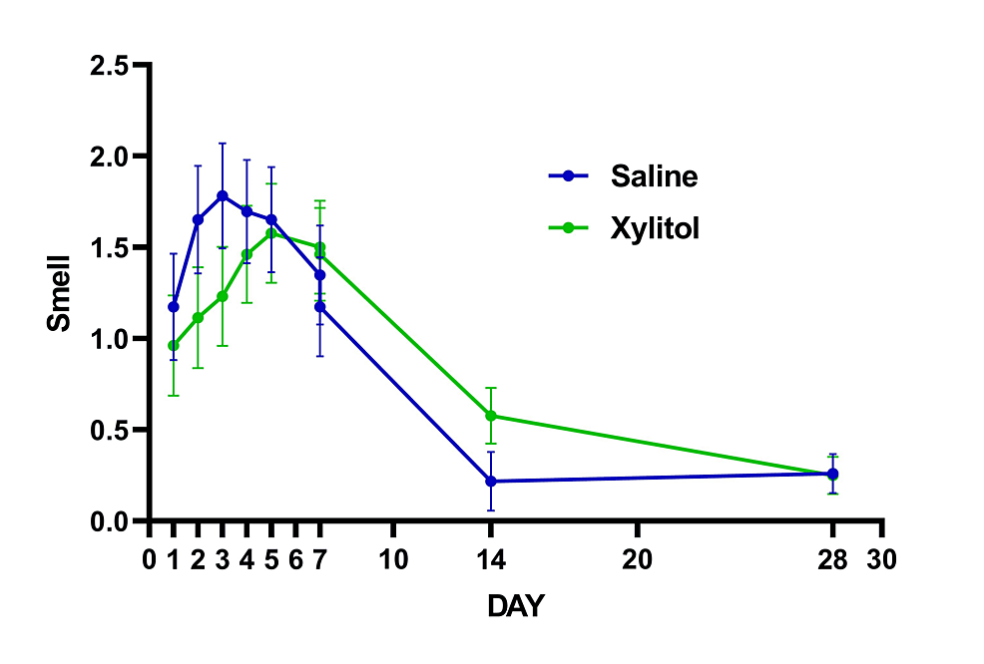
Response to treatment in olfactory function (smell).

Data in graphs are mean ± SEM (standard error of mean) unless otherwise specified. Subjects' characteristics are summarized in Table 2. There were no differences between the treatment groups in any demographic and subject characteristics-related variables, including vaccination status. None of the patients were hospitalized, or required emergency visits in addition to no adverse reactions were reported. There were no statistically significant interactions were found for VAS (P=0.124) (Figure 1), DSS (P=0.448) (Figure 2), and sense of smell (P=0.667) (Figure 3). The proportion of patients reporting nasal congestion was higher (X^2^=5.05; P=0.025) in XYL (73.1%) vs. SAL (41.7%) on Day 4, and on Day 7 (X^2^=5.72; P=0.017) XYL (50.0%) vs. SAL (17.4%). During Day 28 a total of two patients in the SAL had anosmia vs. no patients with anosmia in the XYL, although this difference did not reach statistical significance (X^2^=5.72; P=0.133). 

**Table 2:** Subjects characteristics

Sex	Number of Patients	Past Medical History	Number of Patients	Age (Mean ± SEM)
Male	20	Hypertension	6	
Female	30	Rhinitis	7	
		Diabetes	2	
		Asthma	7	
		Sinusitis	19	
		Vaccination Status	22	
		Smoker	7	
				40.14 ± 2.16

## Discussion

The present study was carried out to evaluate the efficacy of intranasal xylitol formulation to reduce symptoms associated with the SARS-CoV-2 infection. It was rationalized that targeting this novel virus at its main portal of entry and replication point would help ameliorate the symptoms caused by the disease. The COVID-19 clinical manifestations may mimic other infectious and inflammatory conditions affecting the upper respiratory tract. For instance, other viral infections (e.g., influenza, respiratory syncytial virus) or allergic processes may trigger inflammation of the nasal cavity and sinuses, inducing nasal congestion, cough, and anosmia. In the present study, VAS (Figure 1) and the DSS (Figure 2) were used to grade the severity of most common respiratory and systemic symptoms throughout the first 7 days of intranasal therapy and then on days 14 and 28. A pilot study by Weissman et al. reported no significant difference in VAS scores; however, they found improvement in rhinosinusitis symptoms during the xylitol irrigation phase as compared to the saline phase using a crossover design [19]. Conversely, Lin et al. 2017 reported significant improvement assessed by VAS score and sino-nasal outcome test (SNOT-22), as well as a higher level of nitric oxide which is known to enhance local host defense mechanisms, in a group of patients with chronic rhinosinusitis who received xylitol nasal irrigations [27].

In the present study, none of the participants were hospitalized. The findings of this study are somewhat in alignment with those reported by Baxter et al., where rapid initiation of nasal saline irrigation reduced morbidity and mortality in COVID-19 positive outpatients [28]. In older outpatients testing positive for SARS-CoV-2 who initiated nasal irrigations rapidly after diagnosis, the risk of hospitalization or death was eight times lower than national rates reported by the CDC. Moreover, Ciprandi et al. reported that the combination of saline with xylitol and hyaluronate shortened the viral shedding duration in asymptomatic COVID-19 positive subjects [17].

Taken together, the present findings and those of others add to the notion that the nose should be a target for COVID-19 therapies. That is to say, targeting the early infection stages using intranasal agents capable of neutralizing the SARS-CoV-2 may play a vital role in the treatment of COVID-19 for preventing hospitalizations, decreasing mortality, and even slowing down the spread of the virus [6, 7, 15, 17, 29]. Anosmia was one of the main nasal symptoms evaluated due to its high incidence amongst COVID-19 patients, but no significant difference could be determined between treatment groups. However, on day 28, two patients in the SAL group (2/25 = 8%) still reported persistent anosmia, whereas no patients remained with any smelling impairments in the XYL-treated patients. A proposed mechanism by which COVID-19 may induce anosmia describes that the binding of the virus to ACE2 receptors in the olfactory epithelium (OE) stimulates cytokine release and promotes further inflammation and injury [30].

It is important to point out that at least 3-5% of post-COVID patients report persistent anosmia up to six months after initial infection [31, 32]. Hence, the use of intranasal xylitol in the context of early COVID-19 infection may well serve as a preventative treatment to avoid persistent COVID-19-induced anosmia (Figure 3).

Xylitol's exact anti-inflammatory mechanism of action at the nasopharyngeal site may explain the outcome disclosed in this study, with possible effects not only on smell but also taste, given its oral health benefits [33], hence the need to conduct further investigation to determine a correlation between this molecule and olfactory or taste disorders. In addition, the proportion of acute nasal congestion was notably higher in the XYL group (73.1%) versus the SAL group (41.7%) on Day 4, and Xylitol (50.0%) versus saline (17.4%) on Day 7. As a hypertonic and hyperosmolar solution, the Xylitol spray can induce reflex nasal secretions secondary to hyperreactivity of submucosal glands and histamine release [34].

This is study is not without limitations; sample size, the severity of symptoms (excluding severely symptomatic), and intolerance to the xylitol, are to name a few.

## Conclusions

In sum, the present study results show no significant superior benefits in the XYL group over the SAL group regarding the time of symptom resolution, yet, as highlighted before, persistent anosmia was only seen in the saline group. For this reason, xylitol might play a pivotal role at preventing persistent olfactory or even gustatory abnormalities in post-COVID-19 patients. To that end, we encourage further research for a broader comprehension of xylitol's immunomodulatory properties and mechanisms of action at nasopharyngeal epithelium, to aid with COVID-19 management and prevention of sequelae.
